# Supplementary material for: Research on license plate recognition based on graphically supervised signal-assisted training
Source: PeerJ Comput Sci. 2025 Jul 1;11:e2989. doi: 10.7717/peerj-cs.2989 (PMC12453698; doi:10.7717/peerj-cs.2989)
Supplement: Supplemental Information 1 [file peerj-cs-11-2989-s001.docx]

English translations for the Chinese province abbreviations in the CHARS list:

| **Chinese Character** | **English Explanation** |
| --- | --- |
| 云 | Yunnan Province |
| 京 | Beijing Municipality |
| 冀 | Hebei Province |
| 吉 | Jilin Province |
| 宁 | Ningxia Hui Autonomous Region |
| 川 | Sichuan Province |
| 新 | Xinjiang Uygur Autonomous Region |
| 晋 | Shanxi Province |
| 桂 | Guangxi Zhuang Autonomous Region |
| 沪 | Shanghai Municipality |
| 津 | Tianjin Municipality |
| 浙 | Zhejiang Province |
| 渝 | Chongqing Municipality |
| 湘 | Hunan Province |
| 琼 | Hainan Province |
| 甘 | Gansu Province |
| 皖 | Anhui Province |
| 粤 | Guangdong Province |
| 苏 | Jiangsu Province |
| 蒙 | Inner Mongolia Autonomous Region |
| 藏 | Tibet Autonomous Region |
| 豫 | Henan Province |
| 贵 | Guizhou Province |
| 赣 | Jiangxi Province |
| 辽 | Liaoning Province |
| 鄂 | Hubei Province |
| 闽 | Fujian Province |
| 陕 | Shaanxi Province |
| 青 | Qinghai Province |
| 鲁 | Shandong Province |
| 黑 | Heilongjiang Province |
